# Defining Health Movements and Health Needs Across the Life Course: A Qualitative Study

**DOI:** 10.1111/hex.70228

**Published:** 2025-04-10

**Authors:** Alyssa Yenyi Chan, Felicia Jia Hui Chan, Lucas Jia Rong Puah, Muhammad Bin Aman Azamuddin, Priyanka Rajendram, Weng Mooi Tan, Yoek Ling Yong, Zoe Jane‐Lara Hildon

**Affiliations:** ^1^ Saw Swee Hock School of Public Health National University Health System, National University of Singapore Singapore; ^2^ Ministry of Health Office for Healthcare Transformation Singapore; ^3^ Bold at Work Singapore; ^4^ National Public Health and Epidemiology Unit National Center for Infectious Diseases Singapore

**Keywords:** behaviour change, community health movements, formative research, life course approach, population health, Theory‐based programme development

## Abstract

**Background:**

In an effort to improve population health, communities are being enabled to take charge of their health through the Movements for Health (M4H) programme in Singapore. The present study seeks to understand what characterises a health movement and explores health narratives which resonate over the different life stages.

**Methods:**

A multi‐component qualitative formative study was undertaken. Focus group discussions (FGDs, *n* = 12) and one semi‐structured interview (altogether involving *n* = 52 individuals) were carried out with government and community implementation stakeholders, alongside go‐along interviews (*n* = 24, involving 11 volunteers, 13 programme participants) and e‐diaries (*n* = 37, with 22 programme participants and 15 volunteers).

**Results:**

Themes are reported in **bold,** with subthemes in *italics*. Health movement building was defined as an **evolving process** marked by *co‐creation, emotional investment* and *framed by a shared understanding founded on explicit theory*. Furthermore, health movements were characterised **as taking root in the community,** needing *a shared ‘cause’ to* be *self‐sustaining*. They should be able to *garner momentum and be replicable*, and thus ultimately *far‐reaching and inclusive of all walks of life*. Themes cross‐cutting life stages include **concerns about chronic illness**, which are not limited to seniors. **Positive role modelling** is crucial in *encouraging hesitant participants towards healthier behaviours*. Additionally, the **importance of building supportive, emotional connections with implementers** was emphasised. Priority areas for changing health behaviours and informing health literacy planning across various life stages have also been identified. For youths, **mental health** struggles, such as *mood regulation issues,* are prevalent and often exacerbated by *parental invalidation*. Adults tend to deprioritize **social health** due to responsibilities like breadwinning and childminding, *coping through social connections forged among programme participants*. Seniors expressed trepidation regarding their **physical health**, fearing a *loss of independence* and verbalising how *limited mobility affects their ability to exercise and socialise*.

**Conclusion:**

The present study has provided insights into the early phases of the novel M4H community‐led programmatic approach. Our findings defined health movements and health needs across the life course, whilst expanding on related theoretical and applied community development traditions.

**Patient or Public Contribution:**

This study mixes participatory data (i.e. go‐along interviews) with other qualitative data to provide insights into the co‐creation process of health movement building. The study also adopted a user‐centred approach, and the content appropriateness of the programmes was fed back to the community‐level implementers (i.e. Community Movement Champions [CMCs]) and the M4H administrating committee to inform future programming. Future CMCs have taken up the recommendations extracted from stakeholder engagement, where components on sleep, mental health, etc., have been added. Community coaches and commissioning stakeholders are involved in authorship.

## 1 Introduction

### 1.1 Background

Globally, non‐communicable diseases (NCDs) claim the lives of approximately 41 million people per year, totalling 74% of all deaths [1]. Local communities in Singapore are mirroring this trend with an increased prevalence of chronic diseases [2], which is expected to grow alongside our ageing population, greater life expectancy [3] and sedentary behaviours [4]. Furthermore, recent unprecedented global crises have pushed healthcare systems to the brink, highlighting emergent stressors, such as a limited workforce [5] and rising cost of care [6]. Bringing into the foreground the need for health promotion and preventive healthcare management in the community.

**Figure 1:**
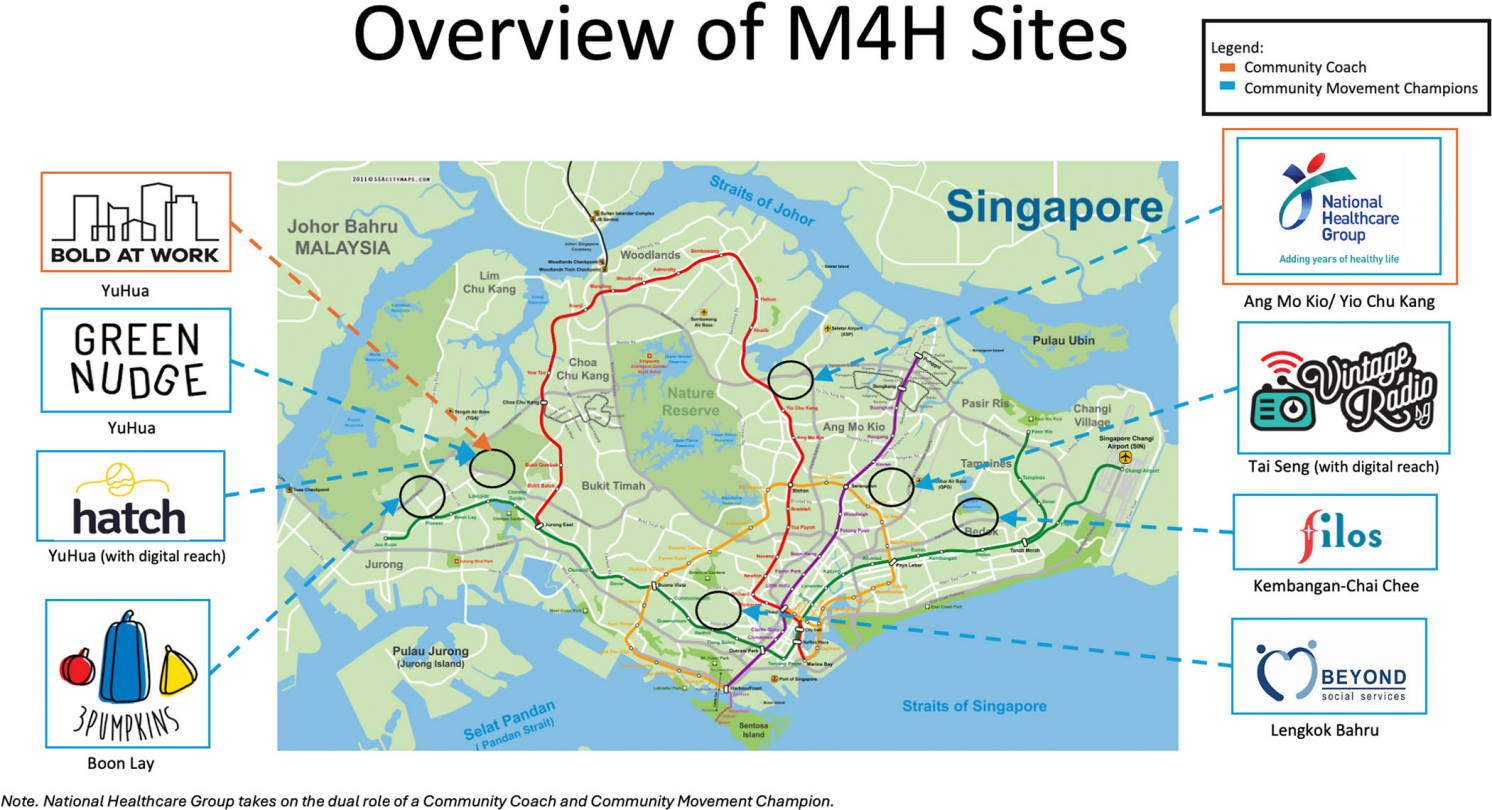
Location of movement building clusters.

In an effort to improve population health, empirical research has advocated for policies endorsing community‐led initiatives [7, 8]. Aligning with the goal of Movements for Health (M4H) [9, 10], a novel programme by the Ministry of Health's Office for Healthcare Transformation (MOHT). Contrasting Singapore's traditional top‐down interventions [11], M4H aims to catalyse neighbourhood‐level community health movements. Figure 1 illustrates the neighbourhoods of which M4H is a part of.

In collaboration with commissioners and community coaching agencies, Community Movement Champions (CMCs) – which include social enterprises and health agencies – develop health programmes tailored to the community's specific needs across different life stages. Coaches skilled in social innovation and health literacy work alongside CMCs to cocreate community‐led, sustainable health movements.

These initiatives aim to empower communities with the skills to foster behaviour change and achieve long‐term impacts. This approach seeks to sow the seeds of bottom‐up community‐led, sustainable, health‐focused programmes through deliberate commissioning structures that provide supportive yet flexible infrastructure. Regular meetings with stakeholders ensure ongoing progress and alignment of objectives.

Movement building expands on asset‐based community development (ABCD) [12] by not only utilising local assets and social capital but also leveraging networks across the M4H programme. This approach capitalises on sharing resources across CMCs, capacity building, and fostering collaboration that extends beyond the neighbourhood level to drive change on a national scale while maintaining the place‐based ethos and participatory element of each initiative.

### 1.2 Theoretical Underpinnings

**Figure 2:**
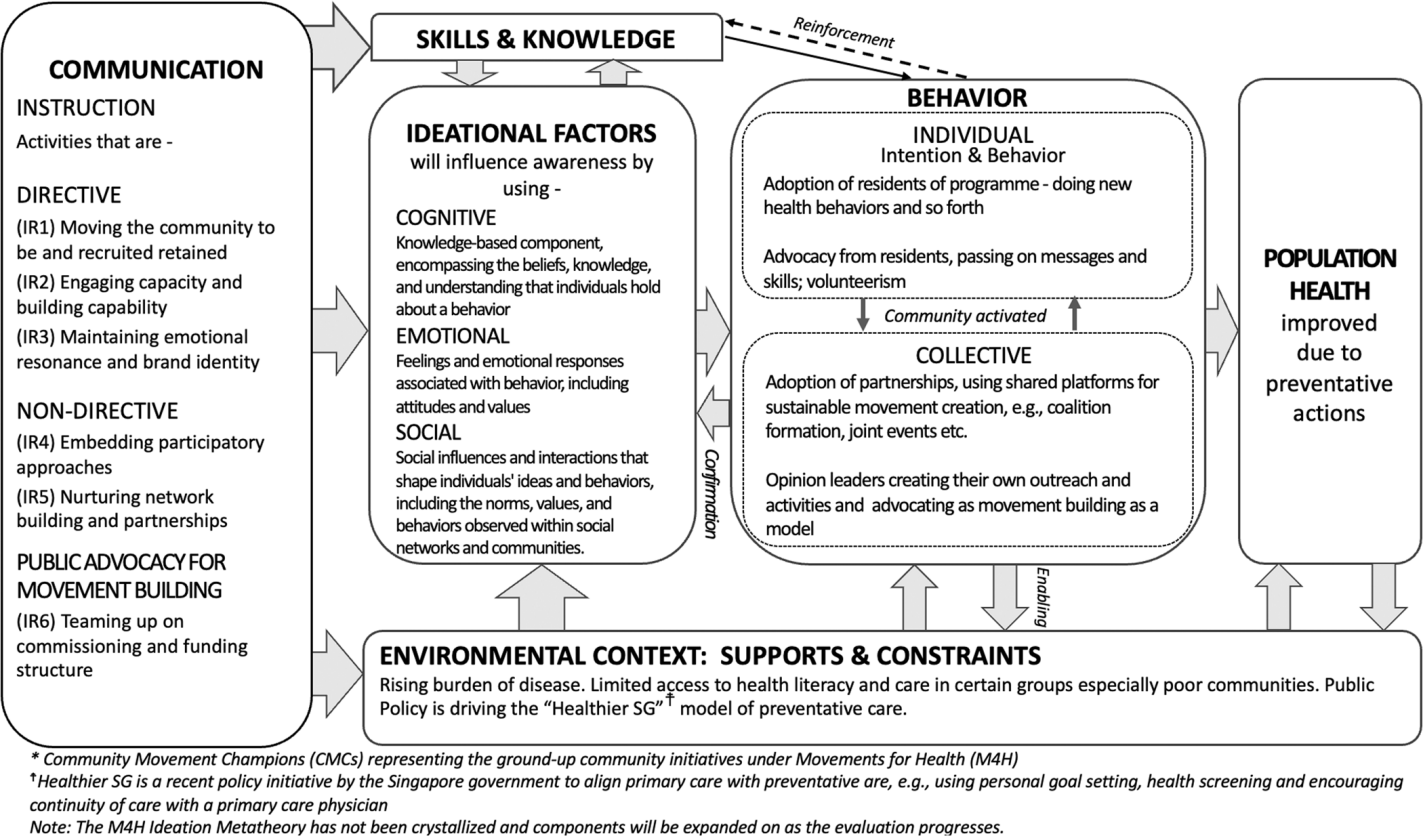
Interim ideation metatheory of the Movements for Health (M4H) Programme.

Paradigms related to coalition building [13, 14, 15], social planning [16] and framing to shift perspectives [17], underscore M4H's programmatic approach. This approach and related planned outcomes are captured by the Ideation Metatheory [18], as outlined in Figure 2. The metatheory proposes a causal pathway, illustrated by the horizontal flow from left to right, which delineates four interconnected stages. Starting with specific communication initiatives that are conceived as Intermediate Results (IR).

IRs are termed as such because the resulting implemented activities are seen as catalysts for longer‐term outcomes, tied to the **3As** as represented in Figure 2. First, by initiating (1) **A**wareness in the form of specific ideations (cognitive, emotional, and social). This, in turn, is seen to lead to (2) **A**doption of the programme, i.e. volunteerism and health behaviours. Ultimately prompting neighbourhood‐level (3) **A**dvocacy for the programme, such as passing on messages and skills that help improve health, or starting new M4H activities and initiatives, thus ensuring its continuity and contributions towards sustained improvements in population health. These stages are influenced by contextual supports and constraints. The vertical ordering of the flow, from top to bottom, reflects an embedded social‐ecological approach [19]. This accounts for the individual, social networks and institutional/societal structures, ending with a focus on the environment.

IRs and related activities (see first box) were designed and developed based on a systematic review, and have been extracted into an implementation tool, termed the Health **MovEMENTs** Checklist [20]. This checklist was created to help guide movement building and to render intervention strategies explicit and thus possible to track and evaluate. The six IRs are described below. These seek to create a sustainable, self‐driven ecosystem that can be nurtured to continually grow and gradually become independent from commissioning structures.

At the community or neighbourhood meso‐level and ultimately seeking to reach the more individual micro‐level, M4H will employ strategies to (1) **Mov**e the community to be recruited and retained in health behaviour change initiatives as well (2) **E**ngage volunteer capacity and capability. All the while emphasising (3) **M**aintaining emotional resonance and brand identity in addition to (4) **E**mbedding participatory approaches. IRs 3 and 4 are seen as *core programme features –* that crosscut all socio‐ecological levels of implementation. At the exo‐level, health agencies (5) **N**urturing network building partnerships and (6) **T**eaming up on commissioning and funding structures are seen to be key to producing sustainable growth of health movements, features that are endorsed at the policy macro‐level.

### 1.3 Study Aim and Research Questions

Based on the above theory‐driven and strategic, evidence‐based planning the present research has been commissioned by M4H with the aim of better understanding the implementation of this novel programme. Specifically, we consider the early stages of the M4H rollout, primarily focusing on IR1, IR3, and IR4. Our research questions are as follows:

1. What characterises a ‘health movement’?

2. What narrative around health resonates over the different stages of the life course?

## 2 Methods

### 2.1 Study Design

**Figure 3:**
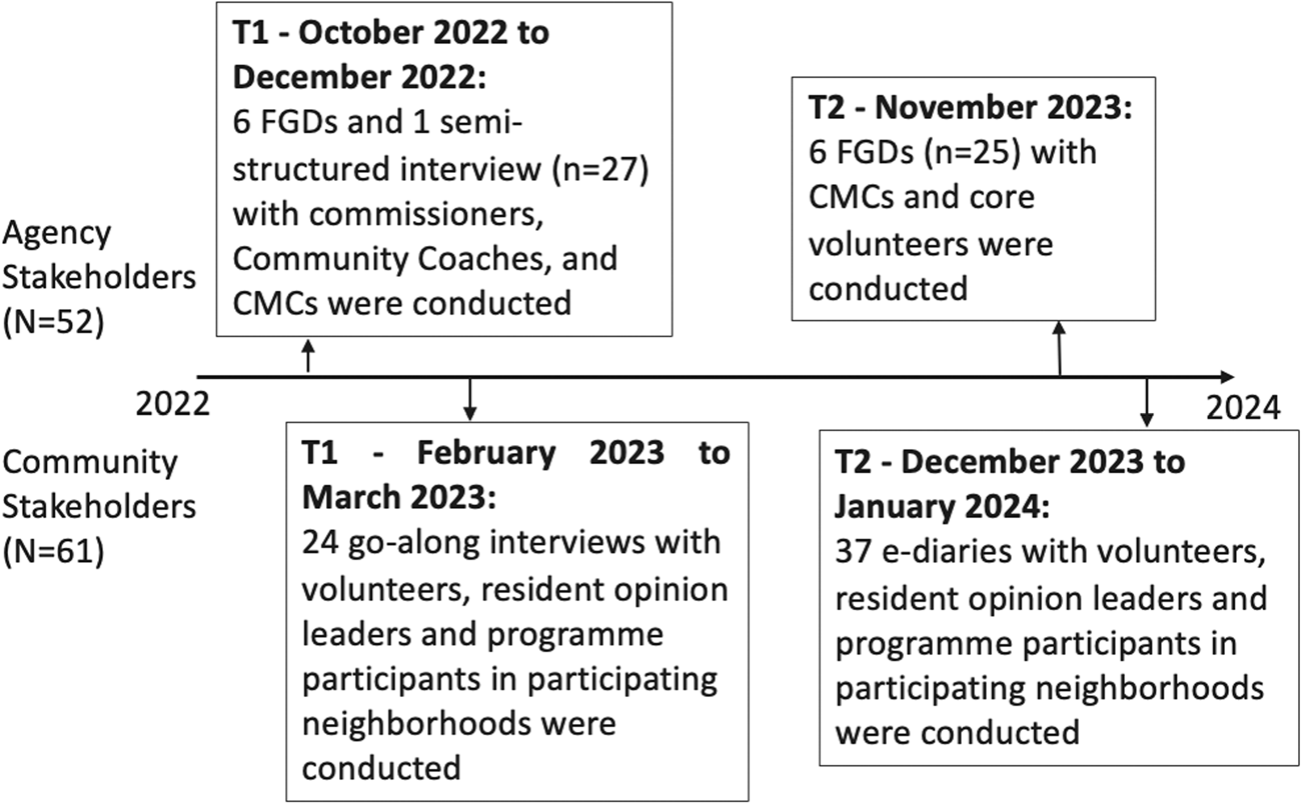
Study's timeline for data collection.

Formative and process‐driven research was carried out following qualitative mixed methods traditions that account for community spaces and perspectives [21]. Methods consisted of yearly stakeholder focus group discussions (FGDs), go‐along interviews, and e‐diaries conducted with a total of *n* = 113 participants, with each method catering to a different target population. Figure 3 illustrates a consolidated timeline for data collection. The methodologies are reported in turn following the COnsolidated criteria for REporting Qualitative research (COREQ) checklist (see Supporting Information S1; [22]).

Two sets of Stakeholder FGDs were conducted on a yearly basis and data are collapsed for analysis in the present study. The first set of these data (*n* = 27 participants) was collected from October 2022 to December 2022, consisting of six FGDs and one semi‐structured interview (March 2023), to include a participant who was unavailable at earlier dates. Another six FGDs were conducted in November 2023, consisting of 25 participants. Stakeholder FGDs were used to engage commissioners, community coaching stakeholders, CMCs, as well as core volunteers. This helped provide a more holistic understanding of the trials and tribulations during the initial stages of implementation [23, 24].

Go‐along interviews (*n* = 24) with volunteers (i.e. individuals supporting the running of the programme) and programme participants from the CMCs were conducted within the respective neighbourhoods' of the CMCs (February to March 2023). This method, also termed ‘walking through spaces’, was selected due to its participatory nature [25, 26]. Anchoring on the neighbourhood setting means that these interviews tapped into resonant narratives that accounted for the environment where the CMCs operate. This provided us with an understanding of different health challenges, resources, and support needs within their neighbourhood.

E‐diaries were also conducted either on‐site or online with volunteers and programme participants of the various CMCs (December 2023 to January 2024). This is an adapted form of the ‘mailbox technique’ which has been used successfully in lower literacy groups [27]. This method seeks to address the problem of how to reach unseen and unheard worlds that otherwise could be out of reach. The data collection itself is underpinned by principles of catharsis, intimate sharing and/or self‐reflection [5, 28].

### 2.2 Research Team and Reflexivity

The research team was led by a senior post‐doctoral qualitative and mixed methods researcher (Z.J.‐L.H.) who led and oversaw the data collection and analysis.

For the FGDs, the data collection team consisted of four graduate and post‐graduate level researchers with experience in qualitative research and backgrounds in Psychology, Public Policy, and Public Health (A.Y.C., F.C., K.X., A.Z.). A.Y.C. served as the key point of contact and liaison for recruitment. Team members would either play the role of facilitator or observer. Field notes were taken by the observer as part of their role.

The team remained consistent for the go‐along interviews (A.Y.C., F.C., K.X.), though an additional graduate researcher, with a background in Public Health was onboarded to support data collection (R.O.). A.Y.C. and F.C. served as points of contact for recruitment. Both points of contact were female in their twenties.

The e‐diary data collection team consisted of consisted of three graduate and post‐graduate level researchers with experience in qualitative research and backgrounds in Psychology, Anthropology, and Public Health (A.Y.C., A.Z., L.P.). A.Y.C. and L.P. served as the key points of contact and liaison for recruitment.

Data collectors were of Chinese, Malay, or Caucasian descent, and consisted of five women and two men. Six members were in their twenties or early thirties, with the other being in their forties. Participants had no prior relationship with the interviewers except for where commissioners or CMCs were involved.

Anonymity was emphasised throughout data collection. Whilst arranging for the interviews, rapport building was initiated through phone calls or WhatsApp messages. Interviewers shared about themselves and reasons for the study and their recruitment, explaining how the study would help understand community‐led initiatives.

### 2.3 Sampling and Recruitment Strategy

**Figure 4:**
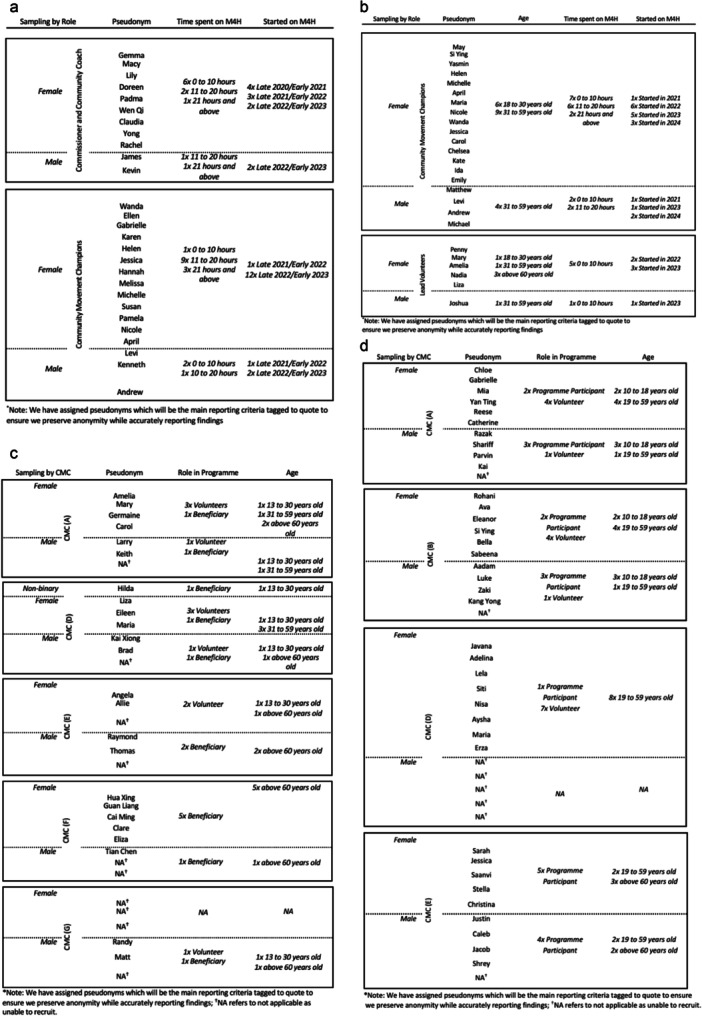
(a). Agency Stakeholder Focus Group Discussion (T1) sampling grid for selection by variation in role in Movements for Health (M4H), *n* = 27*. (b). Agency Stakeholder Focus Group Discussion (T2) sampling grid for selection by variation in role in Movements for Health (M4H), *n* = 25*. (c). Go‐along sampling grid for selection by variation in role in Movements for Health (M4H), *n* = 24*. (d). e‐Diary sampling grid for selection by variation in role in Movements for Health (M4H), *n* = 37*.

Purposive sampling seeking to include all actors involved in the M4H ecosystem was employed. The study team reached out directly to FGD stakeholders and snowballing was used for recruitment into the go‐along interviews and e‐diaries. This yielded a mix of age, occupation and years of experience within the programme. The sampling grid for the first tranche of FGDs is outlined in Figure 4a, second tranche of FGDs is detailed in Figure 4b for the go‐along interviews see Figure 4c, and for the e‐diaries refer to Figure 4d.

### 2.4 Ethical Approvals

Ethics approvals for this study were obtained from the National University of Singapore, Institutional Review Board (IRB), reference number NUS‐IRB‐2022‐131. Informed consent was obtained from all participants.

### 2.5 Data Collection

FGDs were conducted by at least two team members. Zoom was used for online FGDs. Agencies graciously hosted on‐site FGDs in private meeting rooms at their office; the online format did not differ in approach [29]. The FGDs lasted up to two hours. The semi‐structured interview lasted about an hour, though did not prove to be any less in‐depth compared to the FGDs.

Interviews were conducted in‐person on a one‐on‐one basis, except for the pilots, which included paired interviewers including a senior researcher for training purposes. Interviewees were asked to guide the interviewer on a walk around their neighbourhood and introduce places which greatly resonated with them. They were prompted with questions about their views on the programme and uptake of health behaviours as they walked.

Most data collection was undertaken in English. However, for some go‐along interviews, although all participants spoke English, some interviewees preferred to speak in Mandarin (the second most spoken official language in Singapore), which the team was able to accommodate.

All FGDs and interviews were audio‐recorded with the interviewee's permission. The topic guides designed based on the MovEMENTs Checklist [20], for the respective methodologies were piloted and refined after initial interviews, see Supplementary Information S2, S3 and S4 (components used in present analyses are highlighted accordingly).

E‐Diaries were administered both online and on‐site, with participants guided through the online form, mirroring the process for physical copies. Participants were given one week to reflect and complete their entries. For on‐site submissions, a physical mailbox was provided at the CMC's premises and collected after a week. Completion of the e‐diaries took up to 80 min.

Quality checks were performed on each entry to ensure consistency across participants. Follow‐up was conducted on four e‐diaries for clarification. One participant dropped out, and their data was safely discarded. The e‐diaries were structured according to the BioPsychoSocial Model [30], exploring general health understanding and perceptions of change in (1) bio‐physical, (2) psychological and (3) social health. The e‐diaries were piloted and refined accordingly, see Supporting Information S5.

### 2.6 Data Processing and Analyses

Focus group and interview data were extracted using expanded notes [31], relying on detailed summaries while also extracting salient verbatim quotes using audio recordings. We were not able to recontact participants for comments and corrections, although internal cross‐checks were performed on extracted data, and these refined with content agreed within the team.

Online e‐diary data was extracted from Qualtrics and transposed onto an extraction template. For physical e‐diary entries, data was added to the extraction templates within 24 h of data collection. Expanded notes were imported into ATLAS.ti 23 for coding.

In accordance with our stated research questions the data were segmented to focus analyses on programme startup and engaging communities over different stages of the life course. After familiarisation, applied thematic analysis was undertaken, informed by an interpretivist lens [32, 33]. Stakeholder FGDs, Go‐along, and E‐diary data sets were independently coded, and then codes were compared and merged to identify overlapping themes [34].

Data was then merged, meaning qualitative findings were triangulated with other known contextual information shared with the study team, such as insights from email correspondence, meeting documentations as well as our theoretical positionings to help make sense of the data and crystalize the findings.

Coding was undertaken by one analyst (A.Y.C.). Saturation of identified themes was reached in the final stages of coding. Themes were refined, and interpretation was agreed upon based on discussion with a senior qualitative lead (Z.J.‐L.H.) who reviewed the tagged data and assigned codes.

### 2.7 Reporting of Findings

Results are reported according to the sequence of our research questions. Each section summarises major themes in **bold** and their supporting sub‐themes in *italics*. Themes that crosscut a multitude of actors or which are mainly unique to one or the other of these are identified accordingly within the narrative reporting. Illustrative quotes have been reported in tables. Quotes are tagged to assigned pseudonyms (as originally reported in Figure 4a–d) and other relevant framing information.

## 3 Results

### 3.1 Sample Description

A total of 12 stakeholder FGDs and one semi‐structured interview were carried out over two timepoints. The first tranche (T1) was conducted with stakeholders working as a CMC, Community Coach or government‐affiliated programme commissioner, while the second tranche (T2) was carried out with stakeholders working as a CMC or volunteer. Table 1 presents the participants characteristics.

**Table 1:** Description of implementing agency study participants taking part in stakeholder focus group discussion and a semi‐structured interview, *n* = 52.

Sociodemographic information				
Variables	Categories	T1 Interviewees (*N* = 27)	T2 Interviewees (*N* = 25)	Total (*N* = 52)
*Age*	18 to 30 years old	11 (40.7)	7 (28.0)	18 (34.6)
	31 to 59 years old	15 (55.6)	15 (60.0)	30 (57.7)
	Above 60 years old	1 (3.7)	3 (12.0)	4 (7.7)
*Gender*	Female	22 (81.5)	20 (80.0)	42 (80.8)
	Male	5 (18.5)	5 (20.0)	10 (19.2)
*Ethnicity*	Chinese	22 (81.5)	19 (76.0)	41 (78.8)
	Malay	1 (3.6)	3 (12.0)	4 (7.7)
	Indian	2 (7.3)	2 (8.0)	4 (7.7)
	Others	2 (7.3)	1 (4.0)	3 (5.8)
*Time spent on M4H*	0 to 10 h	9 (33.4)	16 (64.0)	25 (48.1)
	11 to 20 h	13 (48.1)	7 (28.0)	20 (38.5)
	Above 20 h	5 (18.5)	2 (8.0)	7 (13.4)
*Role in M4H*	Agency (Commissioners and Community Coaches)	11 (40.7)	0 (0)	11 (21.1)
	Community Movement Champions	16 (59.3)	17 (68.0)	33 (63.4)
	Volunteer	0 (0)	6 (24.0)	6 (11.6)
	Transitioned from Volunteer to Paid Staff Implementer	0 (0)	2 (8.0)	2 (3.9)

The mean age of our interviewees was 38 and ranged from 20 to 72 years old. The majority were female (*n* = 42) and/or of Chinese ethnicity (*n* = 41). More than half the participants were CMCs (*n* = 33) and reported spending a range of four to 40 h on M4H per week, with an average of 11.8 h spent per week. Additionally, a small proportion of interviewees reported transitioning from the role of a volunteer to a paid staff implementer (*n* = 2).

The sociodemographic characteristics of go‐along interviewees are described in Table 2. Similarly, most interviewees were female (*n* = 13) and/or of Chinese ethnicity (*n* = 18). A handful of participants were living in rental flats (*n* = 4), and most had a household income of less than SGD$2000 per month (*n* = 9). We had an almost equal proportion of volunteers and programme participants of *n* = 11 and *n* = 13, respectively. Additionally, quite a few interviewees were retired (*n* = 8).

**Table 2:** Description of programme participants and volunteers taking part in Go‐along Interviews, *n* = 24.

Sociodemographic information		
Variables	Categories	Interviewees (*N* = 24)
*Age*	13 to 30 years old	6 (25.0)
	31 to 59 years old	6 (25.0)
	Above 60 years old	12 (50.0)
*Gender*	Female	14 (58.3)
	Male	9 (37.5)
	Nonbinary	1 (4.2)
*Ethnicity*	Chinese	18 (75.0)
	Malay	4 (16.6)
	Indian	1 (4.2)
	Others	1 (4.2)
*Monthly Household Income*	Below SGD$2000	9 (37.5)
	SGD$2000 to SGD$3999	5 (20.8)
	SGD$4000 to SGD$5999	5 (20.8)
	More than SGD$10,000	2 (8.4)
	Unsure	3 (12.5)
*Accommodation*	Rental Flat	4 (16.6)
	HDB 2 Room	3 (12.5)
	HDB 3 Room	4 (16.6)
	HDB 4–5 Room	7 (29.3)
	Private Apartments	4 (16.6)
	Landed	2 (8.4)
*Occupation*	Employed full‐time	4 (16.6)
	Employed part‐time	8 (33.4)
	Student	3 (12.5)
	Housewife	1 (4.2)
	Retired	8 (33.4)
*Role in Programme*	Volunteer	11 (45.8)
	Programme Participant	13 (54.2)

At the community level, 37 e‐diary entries were collected from either programme participants or volunteers. Participant characteristics are described in Table 3. Similarly, most participants were female (*n* = 25) and/or were of Chinese ethnicity (*n* = 18). The average age of our participants was 33 and ranged from 10 to 68 years old. Most of our participants were single (*n* = 21) and/or living in rental flats (*n* = 12). Close to a third of our participants had a household income of SGD$2000 to SGD$3999 monthly. Most entries were from students (*n* = 16), and slightly more than half were programme participants (*n* = 22).

**Table 3:** Description of programme participants and volunteers taking part in e‐diaries, *n* = 37.

Sociodemographic information		
Variables	Categories	Interviewees Count (%)
*Age*	10 to 29 years old	20 (54.1)
	30 to 59 years old	12 (32.4)
	Above 60 years old	5 (13.5)
*Gender*	Female	25 (67.6)
	Male	12 (32.4)
*Ethnicity*	Chinese	18 (48.6)
	Malay	11 (29.7)
	Indian	8 (21.6)
*Religion*	Christian	11 (29.7)
	Buddhist	2 (5.4)
	Muslim	15 (40.5)
	Hindu	1 (2.7)
	Others	8 (21.6)
*Relationship Status*	Single	21 (56.8)
	Married	12 (32.4)
	Separated/Divorced	4 (10.8)
*Monthly Household Income*	Below SGD$2000	6 (16.2)
	SGD$2000 to SGD$3999	13 (35.1)
	SGD$4000 to SGD$5999	4 (10.8)
	SGD$6000 to SGD$9999	3 (8.1)
	More than SGD$10,000	6 (16.2)
	Unsure	5 (13.5)
*Accommodation*	Rental Flat	12 (32.4)
	HDB 2 Room	4 (10.8)
	HDB 3 Room	2 (5.4)
	HDB 4‐5 Room	9 (24.3)
	Private Apartments	8 (21.6)
	Landed	2 (5.4)
*Education Level*	No formal education	6 (16.2)
	PSLE	8 (21.6)
	GCE ‘O’ Level	2 (5.4)
	GCE ‘A’ Level	3 (8.1)
	Polytechnic Diploma	5 (13.5)
	University Undergraduate	8 (21.6)
	University Postgraduate	5 (13.5)
*Occupation*	Employed full‐time	6 (16.2)
	Employed part‐time	4 (10.8)
	Student	16 (43.2)
	Self‐employed	3 (8.1)
	Retired	5 (13.5)
*Role in Programme*	Volunteer	15 (40.5)
	Programme Participant	22 (59.5)

### 3.2 RQ (1) What Characterises a ‘Health Movement’?

Largely at the agency level, **movement building was portrayed as a process that evolves based on ongoing collective efforts**. Subthemes underpinning these were described as dependent on *consistent co‐creation and emotional investment from the community*. It was emphasised that *consistent exposure and repetition to M4H activities* and messages would help toward movement building.

For some, this was only really able to be seen as ‘concrete’ when *framed by an explicit, evidence‐based theory* with consensus from all actors in the ecosystem on what this would look like. In contrast, one interviewee mentioned that *going with one's gut‐feeling, trial‐and‐error, and even failure is an important part of learning* how to grow a CMC.

Otherwise, **health movements were characterised by taking root in the community,** for instance aiming at being *self‐sustaining*, and being able to build on *a shared ‘cause’*. Also, movements *can garner momentum, and be replicable* across different neighbourhoods. Thus, ultimately, being *far‐reaching and inclusive of all walks of life*.

However, in the context of *implementing initiatives within new communities*, such as schools, there was acknowledgement that the concept of ‘movements’ was *still in the process of being understood*.

Agency stakeholders also emphasised the need for a **cohesive communication, branding and outreach strategy**. Specifically, including *sharing, explicitly what is meant by movement building* within the M4H networks and beyond.

The 3As (i.e. Awareness, Adoption, and Advocacy) were also addressed by agencies and communities alike. At the community level, many described **Awareness** as *gaining buy‐in and generating buzz, currently, this is happening largely by word of mouth*. This is *enticing people to ‘step out’ of their comfort zone*, once the first step is done and enjoyed, this will grow their motivation to want to commit. Agencies defined Awareness as mainly hinging on *having a shared vision –* or even brand identity – around which this buzz could be created.

Correspondingly, community **Adoption** was mainly characterised as *enabling participation in activities and acquiring of new health behaviours*, with some mentioning *increasing volunteerism*. Agencies saw adoption as *driven by partnerships and using shared platforms* within the M4H ecosystem.

As for **Advocacy** at the community level, this was perceived as stemming from *a sense of ownership* from participants, and these being *inspired and enabled to set up their own outreach or activities*, with some ‘passing‐on’ what they learnt to enable health gains in others. *Pass‐it‐on could be a simple gesture of sharing*, such as providing a basic meal to help a neighbour. It can also take the form of *supporting activities that help people be connected*, through informal volunteering, or *passing on learnings to family or friends*, e.g. sharing a health recipe or messages learnt from M4H.

The latter underscores the importance of finding emotionally resonant and relevant health narratives that inspire such types of community‐driven advocacy. Commissioners agree that the key to advocacy is about promoting *movement building as a model*, which entails a cyclical structure of Community Coaches training CMCs and the CMCs working with communities encouraging them to come together and work towards the same goals.

Overarchingly, this definition of ‘health movements’ largely came from the agency level. At the community level, when asked about what makes a ‘movement’ and how this could eventually become self‐sustaining, these ideas did not resonate. Rather, participants spoke about valuing the place‐based and emotional safety of their own neighbourhood‐based activities. Please refer to Table 4 for illustrative quotes on this section.

**Table 4:** Illustrative quotes for defining health movement building.

Themes	Supporting sub‐themes with illustrative quotes
**Movement building was portrayed as a process that evolves based on ongoing collective efforts**	Dependent on consistent co‐creation and emotional investment from the community *‘So what we have done… [is consider] what are the components that they [consultants/researchers] have written that enables a successful movement itself, so kind of take that and [the] scoping review together to look at how then should we shape, what [coaches] will push. How do we then translate that into the emotional resonance?’ –* James, Male, 31 to 59 years old, Agency, Stakeholder FGD T1 Consistent exposure and repetition to M4H activities *‘We will have to repeat some of the engagement or the messages again and again. I think this helps to reinforce some of the messages. So even if we have seen some of the seniors once, twice, in this case, we feel that it's still helpful to reiterate some of the points. So, while it might, for other engagements let's say we use this stuff once or twice, it seems very ‘naggy’. In this case, I think it's helpful to continue to mention it again and again using the same tools, using the same cues. And that's how I think it helps ingrain some of these points‬’. –* Levi, Male, 31 to 59 years old, CMC, Stakeholder FGD T2 Framed by an explicit, evidence‐based theory *‘Stability in terms of like the focusing on like the Theory of Change, the logic model, we have something that we fall back on. We got something that we can anchor on and everybody agrees, this is how we're going to do this and that should stay. But of course, as we go through, we measure, we kind of evaluate and later on make changes’. –* James, Male, 31 to 59 years old, Agency, Stakeholder FGD T1 Going with one's gut‐feeling, trial‐and‐error, and even failure is an important part of learning *‘Everybody say everything is supported by data, yes, but I always believe in a mix of both. Something is an art, other thing is science, but we do need to have the blend of art and science together to actually have something that is more relevant for us’. –* Andrew, Male, 31 to 59 years old, CMC, Stakeholder FGD T1
**Movements were characterised by taking root in the community**	Self‐sustaining *‘I guess a successful movement would then have to be self – sustaining, like in an independent, I guess, momentum that they can drive themselves’. –* M‬acy, Female, 13 to 30 years old, Agency, Stakeholder FGD T1‬‬‬‬‬‬ Building on a shared cause *‘We don't exactly know the specific attributes of what it is but um they mentioned Pinkdot. And basically it's something that is bigger than an individual and it sustains itself, beyond any specific activity because people believe in a cause’. –* Wanda, Female, 13 to 30 years old, CMC, Stakeholder FGD T1‬‬‬‬‬‬ Can garner momentum, and be replicable across different neighbourhoods *‘I think for a movement, it needs to be scalable, and the projects what they do, can be replicated into different areas where maybe it started from a small group, so that needs to also be an existing group and then slowly branch up to the bigger community itself. The momentum that work’. –* James, Male, 31 to 59 years old, Agency, Stakeholder FGD T1‬‬‬‬‬‬ Far‐reaching and inclusive of all walks of life *‘I find it, the word “movement” is something very big…’ –* Melissa, Female, 31 to 59 years old, CMC, Stakeholder FGD T1 In new communities, the concepts of movements is still being understood *‘While we had experienced the primary schools…as the kids progress into adolescence and the relationship between them and the parents as well as towards what excites them, changes quite drastically. So, this is a completely new field for us as well’.‬ –* Matthew, Male, 31 to 59 years old, CMC Stakeholder FGD T2
**Cohesive communication, branding and outreach strategy**	Sharing, explicitly what is meant by movement building *‘For myself, I'm just feeling how important communication would be. And I think it's framing the question or at least framing this information to the respective groups of individuals, volunteers, the users, to seniors is important lah’.‬ –* Levi, Male, 31 to 59 years old, CMC, Stakeholder FGD T2
**Awareness**	Gaining buy‐in and generating buzz – currently, this is happening largely by word of mouth *‘I guess my sensing for some of the work that we've been doing so far to gauge the level of growth has just been fairly simple kind of word of mouth… [this] suggests that there is some level of information sharing around, not so much available on a website, but by words… this person worked with us and they are now reaching out to others. So, I think that kind of give us some level of understanding in terms of how it is growing organically’. –* Levi, Male, 31 to 59 years old, CMC, Stakeholder FGD T1 Enticing people to ‘step out’ of their comfort zone. *‘Once they bring them (participants) out, already improve their health. As long as an individual comes out to walk, the person will be healthy. Better than hiding at home, so the thing is to step out. As long as you are willing to step out, you will be healthy, take the first step. You step out first, people want to go outing, you step out, then you enjoy, then next time you will know how to ‘outing I go!’ – then slowly more lah. Starting you cannot say do this, do that of course’. –* Eliza, Female, 31 to 59 years old, Volunteer, Go‐along Interviews‬‬‬‬‬‬ Having a shared vision *‘Then when we onboard other people right, on a certain cause […] They actually find out because that there's a value that is… important to them and that is attitudes, justice and it's uhm… things like… values. Correct? So that's sort of the sustainable [aspect of what is being done], motivating … people to act’.‬ –* Yong, Female, 31 to 59 years old, Agency, Stakeholder FGD‬ T1‬‬‬‬‬‬ *‘We changed our focus, our own goals [the things] we get out it, but there will be overlapping goals, which is why we choose to do this together’. –* Yong, Female, 31 to 59 years old, Agency, Stakeholder FGD T1
**Adoption**	Enabling participation in activities and acquiring new health behaviours *‘There's ulam raja [ingredients] in the garden… Ya, so that's [how we enable participation in the] movement for them, la. Then it becomes a very natural thing… in an 1‐h time, they can go the garden and go harvest’. –* Michelle, Female, 13 to 30 years old, CMC, Stakeholder FGD T1 Increase in volunteerism *‘So… we also, erm, in terms of outreach; so that you want that interest group, who are higher on the participation ladder. And then there are those who are still eating, basic eating. Even in eating, it's building that culture of hygiene. Like wash your hands, and then after eating what do you do with the plates’.* – Susan, Female, 31 to 59 years old, CMC, Stakeholder FGD T1‬‬‬‬‬‬ Driven by partnership and shared platforms *‘So there was this huge idea of building the ecosystem within the CMC and the belief is that because there was a… kind of a belief that if the CMCs themselves could connect well, and understand each other's assets and resources, that will then spiral to a stronger support or movement network. […] In Jurong as the base, and moving beyond, hopefully’*. – Claudia, Female, 31 to 59 years old, Agency, Stakeholder FGD T1‬‬‬‬‬‬
**Advocacy**	Sense of ownership *‘It also builds up that level of ownership, as a citizen. We should increase our civic – mindedness, and not just… uhm, feel like “Oh, this is something that is not my problem”. If we are serious in being a citizen here, I think we should know that this is our stake and our… if you want to make it a home, it should come from us la’.* – Carol, Female, 31 to 59 years old, Volunteer, Go‐along Interview Inspired and enabled to set up own outreach or activities *‘Our goal is really not to organize the thing for them, but it's really to scaffold and to take small little steps, so that they are confident enough to run on their own. But before you can do, you have to actually work with them and give them opportunities to practice alongside with you until they are confident to run on their own’.‬* – Melissa, Female, 31 to 59 years old, CMC, Stakeholder FGD T1 ‬‬‬‬‬‬ *‘Community has voices. Let the community voice out. Let the community reach out to the organization. So when we say we want to do this, make sure you … help us find donors, because if we can find donors, we wouldn't go straight to you.’* – Maria, Female, 31 to 59 years old, Volunteer, Go‐along Interview‬‬‬‬‬‬ Pass‐it‐on could be a simple gesture of sharing *‘Of course, pass it on is something that we have been doing that in our daily basis. Example, if our food didn't finish, because we want to close down already, so we pass it on la, to our neighbor. If they have, already have food, they pass it on ah, to another family’.* – Maria, Female, 31 to 59 years old, Volunteer, Go‐along Interview Supporting activities that help people be connected *‘The reason why I come up with this fridge is because I want to build relationships with the neighbours, with the new… Newcomers and also instil a good habit, like.. small, small, small parts. Action speaks louder than words, right? So instead of doing movements and say, “this is right, this is wrong” … So what I did is I just have, you know, broccoli and whatever mix that kind of thing, so force that just to take it one, you know, like try, you know, just throw this. And we build relationship as in having a conversation on how we can create a simple recipe for your child. Yeah. So that's how… that's the reason why they.. Every month, they keep on coming back to collect the ration’.* – M‬aria, Female, 31 to 59 years old, Volunteer to CMC, Stakeholder FGD T2‬ Passing on learnings to family and friends *‘So we experiment. […] we learnt a lot from that. So whenever I go out, I will do the same. Pass it on, or teach them, or share with them, on how to do. Uh, how to use a plant, or how to cook with it’. –* Mary, Female, above 60 years old, Programme Participant, Go‐along Interview‬‬‬‬‬‬ *‘So they love learning things. Pizzas was because they learn from uh, a class. And then they learn from it. So they take in the knowledge, they bring it to the [other] kids, they do it for four months’.* – S‬usan, Female, 31 to 59 years old, CMC, Stakeholder FGD T1 Movement building as a model *‘[For the individual level to advocacy, this] has to translate to a community‐led directive of sort, so when many individuals in the community believe in the same thing to work towards, that's when the whole community works towards it, creating a movement’.* – Kevin, Male, 31 to 59 years old, Agency, Stakeholder FGD T1

### 3.3 RQ (2) What Narrative Around Health Resonates Over the Different Stages of the Life Course?

Agencies, in particular commissioners, were keen on targeting and ultimately tracking behaviours over the life course. Therefore, CMCs were selected such that each life stage was represented. The range and content of CMCs is summarised in Supporting Information S6. Observations shared by our study participants showed that health priorities and programme foci differed at the various life stages.

Nevertheless, themes cross‐cutting life stages were found. These pertained to **the need to increase health resources, whilst also making them more accessible**, especially to lower‐income participants, who were viewed as ultimately more likely to end up with multiple chronic conditions.

Activities included cooking and eating together – e.g. *exposure to novel cooking approaches for youth* through providing ingredients and hands‐on learning by doing cooking classes, or *peer‐led low‐impact physical activities for seniors. Uptake was seen as more feasible when healthier alternatives were readily available with little effort*.

*Regular refreshing of fun activities* and *using neighbourhood‐specific digital forum for channelling them* were also heralded as the cornerstone of opening opportunities for participation.

Similarly, **the importance of leveraging positive role modelling,** either *from youth leaders or programme staff leading by example*, also *sharing of stories of resilience, particularly at older ages* was identified as theme cross‐cutting life stages. *These strategies encouraged hesitant programme participants towards new or healthier alternatives* and were mentioned as *helping to build self‐confidence of mentors, as well as mentees*. Emphasis was also placed on *guiding participants to be involved in taking initiative and problem‐solving*.

E‐diary entries also revealed perspectives surrounding health, particularly surrounding social connections and biological functioning. Notably, many participants emphasised **the importance of building supportive, emotional connections with implementers.**

Additionally, most expressed a priority for **maintaining physical activity and mobility**. Though particularly prominent among seniors, *concerns about chronic illness* were not limited to them. Working adults and youth also expressed these concerns, in particular one individual highlighted the misconception that youth are immune to such illnesses simply due to their age.

Tailoring programmes to align with different health priorities at various life stages was perceived as essential to encouraging self‐motivated behaviour change. This will mean planning the right types of health literacy materials, but it starts with mapping how health is internalised. This is unpacked according to life stages below.

#### 3.3.1 Children and Youth (Aged 10 to 20 Years Old)

For children and youth, numerous participants articulated **challenges in defining their own physical health and behaviours**. However, it was noted that with guidance **health behaviour changes were easier to incorporate when taught since young**, in particular, emphasis should be put on *habit formation*. **Youth were also noted to be easily steered to grow into leadership roles.**

**Mental health** struggles such as *mood regulation*, *fatigue*, and a lack of coping mechanisms were the most common dimension of health discussed by interviewees of this age group. Participants also shared frustrations with *invalidation from their parents or authority figures*, e.g. when facing scholastic stress or body dysmorphia.

This extended to youths often *seeking guidance and validation from authority figures*, such as programme implementers and volunteers. In addition, these mental health challenges spilled over into problems with **physical health**. This was largely due to busy schedules, which led to *eating instant meals;* many also *lacked proper sleep routines*.

Positive **social health** outcomes **were** attributed to the *camaraderie fostered during programme activities*. Friends helped to alleviate the perceived burden of mundane tasks and contributed to a sense of social well‐being among participants.

#### 3.3.2 Working Adults (Aged 21 to 59 Years Old)

A dominant theme effecting behaviour change in working adults in our population group was in reference to Maslow's **hierarchy of needs** [35]. Many expressed the importance of *prioritisations for meeting basic needs*, such as having sufficient food.

Making time for **physical health** was seen as low on the list of priorities for many working adults. In this population group, health problems were discussed as underscored *by sedentary lifestyles leading to illness;* one interviewee mentioned concerns about *hereditary diseases*.

Given that the M4H serves many lower‐income populations, physical health was found to be *contingent on the availability of free time*, particularly among working mothers. Concerns about the need for *subsidised food rations to have healthier options* was also highlighted.

Numerous such participants also voiced concerns regarding *poor sleep patterns*. Notably, one of them attributed their sleep disturbances to engaging in mindless scrolling on their phone before bed.

These carried over to **mental health** struggles, with some expressing difficulties in *stress management*. Self‐care was pushed aside as people struggled to *cope with the practicalities of the daily grinds caused by systemic issues*, *such as unstable living circumstances*, *financial and job insecurity*, and worries about related loss of income if illness strikes.

Participants also found it challenging to maintain their **social health**, as they had a duty to *juggle other responsibilities*, such as being the sole breadwinner or child‐minding. Many working adults coped with these challenges by *seeking support and understanding from programme facilitators and volunteers*. Moreover, *social connections forged among participants* themselves also provided upliftment and emotional support.

#### 3.3.3 Seniors (Above 60 Years Old)

Seniors tended to connect health priorities to **nostalgic recollections of their youth**, often prompted by music or shared stories. These *memories served as a motivating force* for them to maintain an active lifestyle. It was notable that such stories of people from the community about resilience or health ‘warriors’ and recovery were used to **highlight the severe consequences of inaction** and *avoiding becoming bedridden*.

From talking to seniors in our study it became clear that these narratives were anchoring onto very real fears related to degrading **physical health** at older ages. Others were concerned about the *affordability of healthcare*. Many feared a *loss of independence*, and some verbalised how *limited mobility affected their ability to not only exercise but also socialise*.

**Mental health** challenges were a less prominent theme but no less salient. Seniors voiced *concern regarding degenerative diseases* but did not feel greatly at risk. However, in terms of their **social health**, many lamented concerns regarding *social isolation* and boredom of being cooped up at home. For some, this was curtailed by *music*, which was *described as a means of catharsis*, particularly when combined with *supportive relationships formed through the CMC programmes*.

Interestingly, discussion of **gender differences** was a dominant theme **raised by seniors**. With many upholding stereotypical views whereby *community activities are seen as more feminine and typically for women*, with *older men feeling awkward or disinterested in these matters*. It was also notable that one individual highlighted the need for *older men to receive more guidance in managing both positive and negative emotions effectively*.

Additionally, a few added on that there was a *tendency to prefer drinking sessions at the local coffee shops to taking part in organised activities*. Some went as far as to describe men as *prioritising drinking sessions at the local coffee shops* over caring for their physical health.

A lesser mentioned but nevertheless important theme was related to **intergenerational interactions**. Incorporating various actors across the life course can be challenging due to *misalignments in interest or schedules*. Further, *younger members may face challenges drawing seniors out of their shell*. However, some noted that *youths bring a new perspective* which nicely complements the experience of seniors. Please refer to Table 5 for illustrative quotes on this section.

**Table 5:** Illustrative quotes for taking a life course approach to behaviour change interventions.

Themes	Supporting sub‐themes with illustrative quotes
**The need to increase health resources, whilst also making them more accessible**	Exposure to novel cooking approaches for youth ‘*Ulang rajaj first time I try it but it goes so well with the pizza…so we try to keep it where the garden start producing, then we help from there’. –* Amelia‬, Female, above 60 years old, Volunteer, Go‐along Interview Peer‐led low‐impact physical activities for seniors ‘*We don't really learn anything there, we just attend events, we don't really learn any skills’.‬ –* Tian Chen, Male, above 60 years old, Programme Participant, Go‐along Interview Uptake was seen as more feasible when healthier alternatives were readily available with little effort ‘*Sometimes they do change like… [our eating habits to become] healthy like some stuff. Sometimes we don't usually eat so much fruits… then like when I do come, I see some, I see a lot of like fruits on the table. […] Ya, more different fruits to try’.‬ –* Germaine, Female, 13 to 30 years old, Programme Participant, Go‐along Interview‬‬‬‬‬‬ Regular refreshing of fun activities ‘*Yes, the walking trails improved our community health. We were all very happy to attend the event. Because we have never been there before, so it's something new, fresh. Like I've said regarding the environment, the environment is really great there. Very good, it's really very good’.‬ –* Cai Ming, Female, above 60 years old, Programme Participant, Go‐along Interview‬‬‬‬‬‬ Neighbourhood‐specific digital forum ‘*So for us, our project is digital user, using digital platforms to promote health and that looks like creating a Facebook group lah, and using the community building features on Facebook to enhance and promote other activities that are the partners talking about’. –* Wanda, Female, 13 to 30 years old, CMC, Stakeholder FGD T1‬‬‬‬‬‬
**Leveraging positive role modelling**	From youth leaders or programme staff leading by example ‘*Then how do I keep myself healthy and positive. That's the part that I share la, mental wellness. Yes, [it will help the listeners] in some ways’.‬ –* Angela, Female, above 60 years old, Volunteer, Go‐along Interview‬‬‬‬ Sharing stories of resilience, particularly at older ages ‘*So uhm, I think basically, they (referring to CMC) wanted to know my journey from the time I started working, everything etc, and all that. And what inspires me to uhm, carry on, especially, at my age, to the seniors’.‬ –* Angela, Female, above 60 years old, Volunteer, Go‐along Interview ‬‬‬‬‬‬‬‬‬‬‬‬‬‬‬‬‬‬‬‬‬‬‬‬ Encouraging hesitant beneficiaries towards new or healthier alternatives. ‘*I think personally for me is when there are role models, who is kind of like their peers, you know, leading the kind of healthy lifestyles and using stories to kind of or narrative to kind of move their hearts towards behavioral change itself and do connect with the why. And that's where perhaps the behavioral change could lah. So I think role model is, is definitely one key part’. –* James, Male, 31 to 59 years old, Agency, Stakeholder FGD T1 *‘Sometimes, you see for example, children ah, they will get one [youth] leader and they will form a football team, to encourage the youngsters ah to play football, so they don't grow haywire’. –* Kai Xiong, Male, above 60 years old, Volunteer, Go‐along Interview Building self‐confidence of mentors, as well as mentees. ‘*They give me a nickname, they call me Albert Einstein, because what you want to do, then just do it, because I feel, why? what's stopping you […] just because you are a child, doesn't mean you can't do it right, you just need some help. They [beneficiaries] initiate it, then I just support it’ –* Keith‬, Male, 31 to 59 years old, Volunteer, Go‐along Interview Guiding participants to be involved in taking initiative and problem‐solving ‘*But also not just presenting solutions for the kids […]. So I think, the other aspect is also the space and the programme's [content]… uhm and how that can better enable children. Y'know? We cannot do that if it's not ongoing, we have to continuously engage the children’. –* N‬icole, Female, 31 to 59 years old, CMC, Stakeholder FGD T1‬‬‬‬‬‬
**Importance of building supportive, emotional connections with implementers**	Children and Youth: ‘*My story is, when I first joined [CMC name], I felt afraid. Then all the teachers and staff welcomed me and cheered me up. They all were very kind and I felt proud being one of the students in [CMC name]’. –* Rohani, Female, 10 to 29 years old, Programme Participant, E‐diary Working Adults: ‘*First, I seek help from [CMC name] so at least I have someone to talk to other than my spouse then one day I was offered a job because back then I was a stay‐at‐home mom, and I was really lazy to start working. I tried a lot of jobs, but nothing works but with [CMC name] I stayed’. –* Javana, Female, 10 to 29 years old, Volunteer, E‐diary Seniors: ‘*They have this wonderful segment of [CMC Programme Name] where listeners get invited onto their programme on air to talk about their lives and get to hear our very own favorite of our choosing. Who else gives you that?’ –* Jacob, Male, above 60 years old, Programme Participant, E‐diary
**Maintaining physical activity and mobility**	Addressing chronic illness concerns *Children and Youth: ‘Young adults can easily have health diseases such as heart disease, diabetes, and high cholesterol. Research shows Heart Attacks are increasing in young adults under the age of 40 […] if they do not take care of their health and diet’. –* Nisa, Female, 10 to 29 years old, Volunteer, E‐diary. *Working Adults: ‘Now that I have 4 grown up young adults and a primary schooling going son, I am diagnosed of diabetic type 2 and high cholesterol. I suspected that this diagnostic is due to my irregular meals while being the main care giver to all 4 of my children back then’. –* Maria, Female, 30 to 59 years old, Volunteer, E‐diary. *Seniors: ‘And lastly, being healthy means you don't have to wake up worrying about what pills you need to take for the day’. –* Justin, Male, above 60 years old, Programme Participant, E‐diary
**Children and youth**	
**Challenges in defining physical health and behaviours**	‘*Uhhhhhhhh, Bro I don't know btw this how I feel PHYSICALLY okay?’ –* Chloe, Female, 10 to 29 years old, Programme Participant, E‐diary
**Health behaviour changes were easier to incorporate when taught since young**	Habit formation in children and youth ‘*So these little habit do you think it's not important? […] It was all started when they say, don't worry when they are older they can start, not true’. –* Amelia‬, Female, above 60 years old, Volunteer, Go‐along Interview
**Youth were noted to be easily steered to grow into leadership roles**	‘*There's a group of them who are growing up a little bit more, reaching 14, going on 15, 16. Uh, we would include them from a certain point of view in our research and also eventually as a [volunteer]’. –* N‬icole, Female, 31 to 59 years old, CMC, Stakeholder FGD T1‬‬‬‬‬‬
**Mental health**	Mood regulation ‘*Personally I think mental health comes first, so maybe yoga programmes, maybe meditation also, to calm their mind, to destress. […] Or some people don't want to destress, they want to vent their anger or something, maybe bring them to this, […] room there is a baseball bat and some stuff to smash with the baseball bat…. let it all out instead of keeping it inside’. –* Hilda, Nonbinary, 13 to 30 years old, Programme Participant, Go‐along Interview‬‬‬‬‬‬ Fatigue ‘*Then Friday, I can stay up till 11.30 max, by the time I go home it's like 10.30 already. Then I will be very drained as well, then I have no mood to do anything, I just want to go and sleep’. –* Brad, Male, 13 to 30 years old, Programme Participant, Go‐along Interview‬‬‬‬‬‬ Invalidation from their parents or authority figures ‘*Mental health I guess, I don't know I guess people just don't know you are suffering from mental health, then they think that it's a joke…Yeah, I don't really talk to adults about my problems, cause like most of them just I guess victim blame’. –* Brad‬, Male, 13 to 30 years old, Programme Participant, Go‐along Interview‬‬‬‬‬‬‬ Sought guidance and validation from authority figures ‘*And I had struggled in mental health before, but if I need to talk to someone, I can talk to adults that work in [CMC name]’. –* Ava, Female, 10 to 29 years old, Programme Participant, E‐diary
**Physical health**	Eating instant meals ‘*The typical eating is eat instant noodles, drinking a lot of gassy drinks. Even, I don't hardly see them eat fruits’. –* Carol, Female, 31 to 59 years old, Volunteer, Go‐along Interview Poor sleep routines ‘*I've tried distracting myself with other things, like sleeping more. I try sleeping more but end up that really messed up my sleeping schedule. Then like I really started like I don't know, I guess sleeping less, then I started getting sleepy in school’. –* Brad‬, Male, 13 to 30 years old, Programme Participant, Go‐along Interview
**Social health**	Camaraderie fostered during programme activities ‘*My friendship from [CMC Programme name] is quite ok, it is because in the kitchen, we will do things together so that we will not take much times’. –* Shariff, Male, 10 to 29 years old, Programme Participant, E‐diary
**Working adults**	
**Hierarchy of needs**	Prioritisations for meeting basic needs ‘*Because (CMC) is doing everything perfect, they give us food rations, they bring out kids for outing, like school holidays, last week they went for outing, they go for SAFRA at Jurong’. –* Liza, Female, 31 to 59 years old, Volunteer, Go‐along Interview
**Physical health**	Sedentary lifestyles leading to illness ‘*But now I realized that in the last five, six years that I have been having this little ailment that has been coming up, which is like a nerve there's a trap nerve or pinched nerve and it actually bothers me every day … I would say that this doesn't happen overnight…it is probably something that I did previously that culminated in this right?‬’* – Thomas, Male, 31 to 59 years old, Programme Participant, Go‐along Interview‬‬‬‬‬‬ Hereditary diseases ‘*I think for me personally, one thing would be genetic illness, because a lot my dad and brother had cancer. So for me, that is the first health concern’.‬ –* Thomas, Male, 31 to 59 years old, Programme Participant, Go‐along Interview‬‬‬‬‬‬‬‬‬‬‬‬‬‬‬‬‬‬‬‬‬‬‬‬‬‬‬‬‬‬‬‬‬‬‬‬‬‬‬ Contingent on the availability of free time ‘*I did try my best to include more fresh foods and vegetables into my diet. I find that when I have more time to cook and a less tight schedule to work around, I do end up gravitating towards very healthy diets’.* – Catherine, Female, 10 to 29 years old, Volunteer, E‐diary Need for subsidised food rations to have healthier options ‘*[No, the neighborhood is not healthy]. Firstly, food rations, do your own research on the food rations. If you want us to stay healthy, for a start, give a proper food ration, not a pathetic food ration. […] Rice, yes, aside from rice, how do you want us to be healthy? [Interviewer: Vegetables, right?] Precisely. […] Just provide these basic [healthy food] for a few days, until we got something to fall back to’.* – Maria, Female, 31 to 59 years old, Volunteer, Go‐along Interview Poor sleep patterns ‘*I usually sleep late because I will be scrolling on TikTok and Instagram before bed’. –* Nisa, Female, 10 to 29 years old, Volunteer, E‐diary
**Mental health**	Stress management difficulties ‘*Cause it's either I stress‐eat or I got no appetite, there is no middle. Which is why a healthy mind (for me at least) is important. If your mind is in it, then the road is more bearable’.* – Adelina, Female, 10 to 29 years old, Programme Participant, E‐diary Coping with practicalities of the daily grinds caused by systemic issues ‘*Some race ah, the parents can't even take care of themselves, then they produce 3, 4, 5 children. Because the house is 1 room, 1 home. […] So where you expect them to sleep? So … night time they cannot sleep, so how can you expect them to go to school? So it becomes the children are affected… so this poverty ah, past from one generation to another’.* – Kai Xiong, Male, above 60 years old, Volunteer, Go‐along Interview‬‬‬‬‬‬‬ Unstable living circumstances ‘*I witness families went through depression just for being in a care giving roles. This depression impacted the women in every household because of of the expectation she put up for herself’.* – Maria, Female, 30 to 59 years old, Volunteer, E‐diary Financial and job insecurity ‘*While eating a balanced diet will help with many factors of physical and emotional health for the long term, if one is concerned or struggling about finances, the opportunity to eat this balanced way may be challenging’.* – Kai, Male, 30 to 59 years old, Volunteer, E‐diary
**Social health**	Juggling other responsibilities ‘*Ya they (single parents) worried because they need to work hard to give their kids to eat, they need to pay for rental flat, they need to pay for bill, everything bill, then the school bill, so they need to work hard, that's why they don't care about their health, even they never go checkup, when they fall sick, and go see the doctor, then a lot of sickness came up. This is the very hard part for them. Because we go through a lot of this in the rental flat’.*‬ – Liza, Female, 31 to 59 years old, Volunteer, Go‐along Interview‬ Support from programme facilitators and social connections among participants provided emotional support ‘*All the staff very friendly n they like my family too..[CMC name]is very helpful and also a good listener to family that need someone to talk’.* – Siti, Female, 30 to 59 years old, Volunteer, E‐diary Social connections forged among participants ‘*We have an ongoing groups for YOUNG MOTHERS. […] Usually we do meet ups and celebrates some holidays together to do some potluck’.* – Javana, Female, 10 to 29 years old, Volunteer, E‐diary
**Seniors**	
**Nostalgic recollections of youth**	Memories served as a motivating force ‘*I must say [CMC name] can be bittersweet like some Shakespearean novel. We all play certain Acts in our lives that could not be recreated except through music on [CMC name]’.* – Justin, Male, above 60 years old, Programme Participant, E‐diary
**Highlight the severe consequences of inaction**	Avoiding being bedridden ‘*Tell them that if they want to save money and not go and see the doctor, go and exercise. If you want to er die in good shape, go and exercise. Don't become a bedridden’.* – Raymond, Male, above 60 years old, Programme Participant, Go‐along Interview‬‬‬‬‬‬
**Physical health**	Loss of independence ‘*I mean really it's no joy. I've seen many people, I mean live to a ripe old age. You want to live to a ripe old age, then er but with all these tubes and everything and lying in bed and having a nurse or a helper help you, I don't want that. I've seen some and they survive for years and years, and it costs a lot of money for one thing, and another thing is you have no quality of life, so what is that’.* – Raymond, Male, above 60 years old, Programme Participant, Go‐along Interview‬‬‬‬‬‬ Limited mobilised affected their ability to not only exercise but also socialise ‘*I guess it depends on where we are going to walk, how long are we going to walk. Because right now I also have an issue with my backbone, my vertebrae […]. I do walk, I go on a treadmill for about half an hour, I guess I can walk for some time like half an hour, 1 hour, but when the pain comes then I want to stop’.* – Raymond, Male, above 60 years old, Programme Participant, Go‐along Interview
**Mental health**	Concern regarding degenerative diseases ‘*Thankful I do not have dementia or Alzheimer's and thankfully, none of my family, I mean my extended family, meaning my siblings, none of them have it also…of course it concerns me, but they say that if you have dementia or Alzheimer's, you don't know what I happening around you. So you're a vegetable, worse than a vegetable, maybe you can walk around, but you don't know where you're going or what you have done’.‬* – Raymond, Male, above 60 years old, Programme Participant, Go‐along Interview‬‬‬‬‬‬
**Social health**	Social isolation ‘*I think that's very important because a lot of people when they retired or become unemployed or reach the age that they have to go, they just sit back and relax and that's it, you know. And then as a result of which your body will deteriorate faster and then yeah, it just goes downhill. You deteriorate very fast you see’.* – Raymond, Male, above 60 years old, Programme Participant, Go‐along Interview‬‬‬‬‬‬ Music was also described as a means of catharsis ‘*If the dance floor is available at an event, you will notice it's us old folks that will hit the dance floor first. We will jive and swing, and we will end up exercising our bodies to the music. It has been said that music is like medicine to the mind and soul. Dance keeps the body agile and moving. All these help you to remain healthy both in mind and body’.* – Caleb, Male, 30 to 59 years old, Programme Participant, E‐diary Supportive relationships formed through the CMC programmes ‘*It is difficult today to find a platform for seniors to gather and find friends. At [CMC name], we see a connection to the past and friendships are formed. This bonding will keep us active and give us an excuse to remain fit so that we can sing and dance together at the next [CMC name] event or gathering’.* – Caleb, Male, 30 to 59 years old, Programme Participant, E‐diary
**Gender differences raised by seniors**	Community activities are seen as more feminine and typically for women with older men feeling awkward or disinterested in these matters ‘*He doesn't want to because he's a man. Besides, most of these activities is catered towards women, I mean walking trails and excursions are for women. In a group of 20, there are only about 2 men anyways. There are very few men’.*– Guan Liang, Female, above 60 years old, Programme Participant, Go‐along Interview‬‬‬‬‬‬ Tendency to prefer drinking session at the local coffee shops to taking part in organised activities ‘*For example, some of the older folk living here, they will gather at the local coffeeshop to drink daily. The coffeeshop is their own “Community Center”, they meet there for food, drinks, and catch‐ups. For us men, that is our interest. Asking them to go exercise isn't really something that would be interesting to them’.‬* – Tian Chen, Male, above 60 years old, Programme Participant, Go‐along Interview‬‬‬‬‬‬‬ Older men to receive more guidance in managing both positive and negative emotions effectively ‘*I could listen to the music with an open mind and take in the good and the bad, whilst previously I would avoid listening to a particular song because I was afraid of the emotions it would precipitate’.* – Justin, Male, above 60 years old, Programme Participant, E‐diary‬‬‬‬‬‬
**Intergenerational interactions**	Misalignments in interests ‘*I don't think the younger generation would be interested in attending activities led by seniors, […]. The youth will think it's old fashioned and are disinterested in what matters to seniors. Especially my grandchildren, they find it boring, so boring. They won't have any interest. I don't think my kids would be interested either, they have their own pattern. It's just easier to relate to people in the same generation as us’. –* Guan Liang, Female, above 60 years old, Programme Participant, Go‐along Interview‬‬‬‬‬‬ Misalignments in schedules ‘*They have their own things to do, like they have tuition or co‐curricular activities. So, they have their own schedules, and it can be hard to find a time that suits everyone’. –* Tian Chen, Male, above 60 years old, Programme Participant, Go‐along Interview‬‬‬‬‬‬ Younger members may face challenges drawing seniors our of their shell ‘*So when I talk to them, they are like dry texters, like what am I supposed to continue the question with. […] they talk like 1 to 2 sentences then done. […] like it is always me to continue the conversation la, like why can't it be both. So it is hard, but slowly I can adapt to it’. –* Matt, Male, 13 to 30 years old, Volunteer, Go‐along Interview Youths bringing a new perspective ‘*We will exchange notes lor. Because the perspective is different right? She is a young lady, uh uh uh, doing similar project, not exactly, but similar projects for seniors right? I'm a senior doing project for uh seniors. So when we talk, we learn from each other la, because perspective is different the approach is a little bit different la’. –* Randy, Male, above 60 years old, Programme Participant, Go‐along Interview

## 4 Discussion

### 4.1 Implications

This formative study was undertaken with the purpose of building on our Ideation Metatheory, clarifying our understnding of community involvement in M4H, behaviour change processes, and ultimately informing programme evaluation. We present a consistent definition of health movements and demonstrate different health needs tailored to each life stage.

Health movement creation, albeit similar to ABCD in utilising community assets and social capital [12, 36, 37], sets itself apart by emphasising self‐sustenance through advocacy (e.g. passing on learnings to enable health gains for others) and replicability across diverse neighbourhoods (e.g. sharing of implementation tools and protocols). Clearly distinguishing and cementing the definition of health movements and the core elements of its theory base from existing community approaches [12], allows for creating shared understanding of the programme aims, overlapping goals between CMCs, and sharing of innovation and resources across the various actors in the M4H ecosystem.

Presently, movement‐building initiatives appear to leverage well‐connected individuals to facilitate the adoption of new activities. In the long run, sustainable movements require activating and connecting entire communities to new opportunities for health.

Movement building as a model thrives on robust networks and partnerships among agencies and community members collaborating to enact positive change within their communities. Furthermore, equipping practitioners, policymakers, and funders with the nuanced processes underpinning movement building, future commissioning and funding structures can better accommodate community‐led initiatives.

Drawing upon literature on eliciting community health needs across the life course [38], our study has also highlighted communities' perspectives and experiences of health at different life stages.

Local literature on community‐led intiatives is limited, and where available has largely focused on community dwelling seniors. [21, 39]. Otherwise, dialogues on health priorities tend to be done with at‐risk individuals, such as seniors in care homes [40], or youths facing mental health crises [41]; working adults priorities tending to be most under‐researched in either setting. Thus, our study contributes to a better understanding of health promotion through outlining which aspects of biopsychosocial health were a priority and why at different life stages, here, in Singapore communities.

Furthermore, M4H is a large programme that spans across neighbourhoods, we are cognizant that there are complex ethnic variations to account for, beyond language. Based on our purposively selected sample, the data did not reflect ethnic differences across the thematic analyses. However, what does bind our population is similarities found in lower income groups, compared to those in a higher socioeconomic income bracket. For instance, related themes came up for working adult groups (e.g. prioritising for meeting basic needs, such as having sufficient food) and seniors (e.g. concerns about the affordability of healthcare).

Ultimately, the M4H programme and its formative research were initiated to address a gap in the literature on community development strategies in upper‐middle and high‐income Asian countries, like Singapore, where empirical studies on ‘community empowerment’ and ‘community‐led initiatives’ are limited [42]. Existing research predominantly focuses on health services in non‐Asian contexts [43, 44], which differ in health priorities and community structures. The M4H initiative thus aims to develop self‐sustaining health movements, especially relevant given rising healthcare costs and a growing emphasis on health promotion.

### 4.2 Recommendations

The facilitation of movement building is underscored by the imperative of using an agreed upon definition. This will be central to sustaining a shared vision and sharing of resources such as a centralised repository of health literacy information and implementation tools that can further empower agencies, volunteers, resident opinion leaders, and community members.

Furthermore, this shared definition allows for future justification for funders to be open to having flexible funding requirements, rather than the typical strict Key Performance Indicators (KPIs). Allowing the community to be hands‐on means there needs to be some open‐ended outcomes in their project deliverables. We recommend that initial KPIs for newer CMCs focus on measuring processes and participation in capability building or running of participatory events to develop, test the emotional resonance of, and grow programmes.

Given the novelty of M4H a multi‐media campaign sharing the principles of health movement building within and across neighbourhood is also recommended. Such a campaign would raise awareness of health literacy as a collective journey that requires addressing health needs at different life stages of the life course using community‐led approaches and the advantages of collectives that span beyond one's immediate neighbourhood.

Tailoring programmes to match different health priorities involves planning appropriate health literacy materials that align with how individuals internalise health, ultimately leading to self‐motivated behaviour change. In addition, by embracing collectivist thinking, leveraging social networks and adopting a family‐oriented approach communities can play a role in shaping both their own health trajectories and influencing population health outcomes.

## 5 Strengths and Limitations

The strength of this research lies in its strong theoretical underpinning which helped us to direct the collection and analysis of a very large body of data. We were also well connected to the CMCs and the community. CMCs and study participants were aware that the data would be used to assess their programmes, thus we were aware of the risk of social desirability. The team did their best to override this through reassuring study participants about confidentiality and positioning the study as an anonymous quality improvement initiative.

Additionally, due to the complexity and practical constraints of this intervention, we were unable to track changes in individual health priorities over time. However, our approach captures a comprehensive snapshot of health priorities across various life stages, offering a valuable understanding of life‐stage health needs within today's health landscape. We would argue the present findings ring true to early experiences on the ground, though dynamics have also since evolved. Ongoing, future longitudinal analyses are planned to track changes over time.

## 6 Conclusion

The present study has provided insights into the early phases of the novel M4H community‐led programmatic approach. Guided by the MovEMENTs Checklist [20], our findings define health movements and the various health needs across the different life stages, whilst expanding upon the longstanding related theoretical [13, 14, 15, 16, 17] and applied [12] community development traditions. The present study also contributes to our understanding of constructing self‐sustaining movements and empowering communities towards healthier behaviours.

## Author Contributions

**Alyssa Yenyi Chan:** writing – original draft, data curation, formal analysis, investigation, validation, visualisation, project administration. **Felicia Chan Jia Hui:** data curation, investigation. **Lucas Puah Jia Rong:** data curation, investigation. **Muhammad Azamuddin Bin Aman:** data curation, investigation. **Priyanka Rajendram:** resources, funding acquisition. **Weng Mooi Tan:** resources, funding acquisition. **Yoek Ling Yong:** funding acquisition, resources. **Zoe Jane‐Lara Hildon:** conceptualisation, methodology, software, writing – review and editing, funding acquisition, supervision.

All authors commented and agreed on the final manuscript prior to submission.

## Ethics Statement

Ethical approval for this study was obtained from the National University of Singapore's Institutional Review Board, Singapore (Reference No. NUS‐IRB‐2022‐131).

## Consent

All participants gave e‐consent before data collection.

## Conflicts of Interest

The authors declare no conflicts of interest.

## Supporting information

Supporting information.

Supporting information.

Supporting information.

Supporting information.

Supporting information.

Supporting information.

## Data Availability

The data sets used are not available to preserve anonymity as per the study team's commitment during institutional ethical review.
