# Supplementary material for: Defining Health Movements and Health Needs Across the Life Course: A Qualitative Study
Source: Health Expect. 2025 Apr 10;28(2):e70228. doi: 10.1111/hex.70228 (PMC11983323; doi:10.1111/hex.70228)
Supplement: Supplementary file 1 — Supporting information. [file HEX-28-e70228-s003.docx]

COREQ (COnsolidated criteria for REporting Qualitative research) Checklist

A checklist of items that should be included in reports of qualitative research. You must report the page number in your manuscript where you consider each of the items listed in this checklist. If you have not included this information, either revise your manuscript accordingly before submitting or note NA.

| **Topic** | **Item**  **N^o^** | **Guide, question description** | **Page reported** | **Notes** |
| --- | --- | --- | --- | --- |
| **Domain 1: Research team and reflexivity** | | | | |
| *Personal characteristics* | | | | |
| Interviewer/facilitator | 1 | Which author/s conducted the interview or focus group? | p. 5 | This information is condensed under the methods section, subheading “research team and reflexivity”. |
| Credentials | 2 | What were the researcher's credentials? e.g. PhD, MD | p. 5 |  |
| Occupation | 3 | What was their occupation at the time of the study? | p. 5 |  |
| Gender | 4 | Was the researcher male or female? | p. 5 |  |
| Experience and training | 5 | What experience or training did the researcher have? | p. 5 |  |
| *Relationship with participants* | | | | |
| Relationship established | 6 | Was a relationship established prior to study commencement? | p. 5, 6 | A hybrid model of data collection was adopted, rapport building efforts are described. |
| Participant knowledge of  the interviewer | 7 | What did the participants know about the researcher? e.g. personal goals, reasons for doing the research | p. 5, 6 |  |
| Interviewer characteristics | 8 | What characteristics were reported about the inter viewer/facilitator? e.g. Bias, assumptions, reasons and interests in the research topic | p. 5, 6 |  |
| **Domain 2: Study design** | | | | |
| *Theoretical framework* | | | | |
| Methodological orientation  and Theory | 9 | What methodological orientation was stated to underpin the study? e.g. grounded theory, discourse analysis, ethnography, phenomenology, content analysis | p. 7 | This study utilised applied thematic analysis and interpretivism. |
| *Participant selection* | | | | |
| Sampling | 10 | How were participants selected? e.g. purposive, convenience, consecutive, snowball | p. 5, 6 | All interviewees were approached via WhatsApp, and the research team was not met with any rejections or refusals to participate. However, there was one drop outs reported on page 7. |
| Method of approach | 11 | How were participants approached? e.g. face-to-face, telephone, mail, email | p. 5 |  |
| Sample size | 12 | How many participants were in the study? | p. 7, 8 |  |
| Non-participation | 13 | How many people refused to participate or dropped out? Reasons? | p. 6 |  |
| *Setting* | | | | |
| Setting of data collection | 14 | Where was the data collected? e.g. home, clinic, workplace | p. 6, 7 | Setting for interviews was described. Presence of observers for the FGDs are also reflected. Important characteristics are included in Table 1a to 1c. |
| Presence of non- participants | 15 | Was anyone else present besides the participants and researchers? | p. 5 to 7 |  |
| Description of sample | 16 | What are the important characteristics of the sample? e.g. demographic data, date | p. 7, 8 |  |
| *Data collection* | | | | |
| Interview guide | 17 | Were questions, prompts, guides provided by the authors? Was it pilot tested? | p. 6, 7 | Topic guide sections are listed in the manuscript, while the tool itself is shared in a supplementary file 2 to 5.  Four e-diaries were followed-up on to clarify content.  Field notes were taken by observers of the FGD.  Largely due to ethical constraints/ preservation of anonymity we were not able to recontact participants for comment and corrections. |
| Repeat interviews | 18 | Were repeat interviews carried out? If yes, how many? | p. 6, 7 |  |
| Audio/visual recording | 19 | Did the research use audio or visual recording to collect the data? | p. 6, 7 |  |
| Field notes | 20 | Were field notes made during and/or after the interview or focus group? | p. 6 |  |
| Duration | 21 | What was the duration of the interviews or focus group? | p. 6, 7 |  |
| Data saturation | 22 | Was data saturation discussed? | p. 7 |  |
| Transcripts returned | 23 | Were transcripts returned to participants for comment and/or correction? | p. 7 |  |

| **Topic** | **Item**  **N^o^** | **Guide, question description** | **Page**  **reported** | **Notes** |
| --- | --- | --- | --- | --- |
| **Domain 3: analysis and findings** | | | | |
| *Data analysis* | | | | |
| Number of data coders | 24 | How many data coders coded the data? | p. 7 | Methods of analysis are fully described and tied to methodological orientation and theory as introduced above.  Largely due to ethical constraints/ preservation of anonymity we were not able to recontact participants for feedback. |
| Description of the coding tree | 25 | Did authors provide a description of the coding tree? | p. 7 |  |
| Derivation of themes | 26 | Were themes identified in advance or derived from the data? | p. 7 |  |
| Software | 27 | What software, if applicable, was used to manage the data? | p. 7 |  |
| Participant checking | 28 | Did participants provide feedback on the findings? | p. 7 |  |
| *Reporting* | | | | |
| Quotations presented | 29 | Were participant quotations presented to illustrate the themes/findings? Was each quotation identified? e.g. participant number | p. 9, 12 | We judge that consistency is illustrated between identified themes and quotes. Themes for the three IRs of focus are summarised in Table 2 and 3. Major themes and minor themes have been described in-text with major themes in **bold**, their supporting subthemes in *italics*. |
| Data and findings consistent | 30 | Was there consistency between the data presented and the findings? | p. 8 to 12 |  |
| Clarity of major themes | 31 | Were major themes clearly presented in the findings? | p. 8 to 12 |  |
| Clarity of minor themes | 32 | Is there a description of diverse cases or discussion of minor themes? | p. 8 to 12 |  |

*Developed from: Tong A, Sainsbury P, Craig J. Consolidated criteria for reporting qualitative research (COREQ): a 32-item checklist for interviews and focus groups. International Journal for Quality in Health Care. 2007. Volume 19, Number 6: pp. 349 - 357*

*Once you have completed this checklist, please save a copy, and upload it as part of your submission. DO NOT include this checklist as part of the main manuscript document. It must be uploaded as a separate file.*
