# Supplementary material for: Defining Health Movements and Health Needs Across the Life Course: A Qualitative Study
Source: Health Expect. 2025 Apr 10;28(2):e70228. doi: 10.1111/hex.70228 (PMC11983323; doi:10.1111/hex.70228)
Supplement: Supplementary file 2 — Supporting information. [file HEX-28-e70228-s006.docx]

TOPIC GUIDE

**Stakeholder FGDs with implementors: Process Evaluation Year 1**

**Broad research questions:**

1. What narratives around health and collective wellbeing, whether direct or indirect, are resonating with the community?
   1. MOHT: Why is M4H needed? [what is its mission]
   2. CMCs: How do CMCs conceive of their “story” – the reason they wanted to create their start-up? [what are the self-conceived missions for the CMC]
2. How can the key actors in the ecosystem best mine and intervene on the resonant narratives within the community?
   1. What implementation processes are likely to work – building in IRs.

**Icebreaker**

To introduce yourselves we can play a little game – please tell me ***one word*** that best defines how your implementation is going so far: e.g. Chaotic; Rocky; Fantastic etc. discuss why they chose this.

**CONTENT DEVELOPMENT FOR M4H**

1. **Emotional resonance maintaining consistent branding/ positioning that resonates with the community**

- What is ‘your story’ - or the main reason for setting up the programme?
  - How does this cater to community needs?
  - In what ways do you think there is “emotional” resonance in the way you are positioning your project?
  - Engage with the spaces in your community?
- Do you think the types of things on offer in the programme would lead to changing people’s behavior?
  - In what ways? Why or why not?
  - What aspects of health have you ended up most focused on? Why?
  - What about different stages of life?

1. **Participatory engagement [IR4]**

- How do you involve people in choosing how the programme will run and what they will look like?
  - How are programme leaders from the community being identified and persuaded to help run things? Perhaps as volunteers?
  - What in your view makes the way you run the programme into a ‘movement’, which eventually could be self-sustaining?

**OUTREACH AND GETTING STARTED**

1. **Demand generation**

- How has your outreach been so far?
- How has covid-19 affected things?
- What are your views on getting beneficiaries to “pledge their commitment”? [i.e., sign up formally to pledge to be volunteers]
- How do you plan to recruit beneficiaries?
- Can you tell me about the people that you anticipate will want to attend your activities?
- What stage of life or ages do they tend to be, and how does this affect the way you run things?
- What could make your target group want to get and stay involved?
- What kind of person might want to join with a little persuasion [hard to reach populations], but not immediately interested in taking part? What kind of things might be persuasive to them?

1. **Capability – growing volunteer knowledge**
   - How has the coaching / training events with Bold community coaching been going?
   - What training needs have you identified for future?
   - How do you plan to train your volunteers?
2. **Capacity – building networks and partnerships**

- Do you think you can find community members keen to run their own activities in the programme? [how do you motivate people to run their own activities?]
- What kind of outreach works best to get people interested in taking part in things so far?
- Do you think it is feasible to run activities where participants “pass on” or teach about things they learnt to others which they learnt through the programme? Why/why not?
- Are you planning to partner with anyone? Are any such collaborations currently happening?
- What makes people want to collaborate?

**COMMISSIONING AND FUNDING**

- How do you see MOHT’s role in supporting your project develop?
- Do you have any plans to supplement your funding?

**Potentially relevant topics reviewed to supplement present analyses.**

***ANYTHING TO ADD**

- Do you have anything to add?

*The End.*
