# Supplementary material for: Defining Health Movements and Health Needs Across the Life Course: A Qualitative Study
Source: Health Expect. 2025 Apr 10;28(2):e70228. doi: 10.1111/hex.70228 (PMC11983323; doi:10.1111/hex.70228)
Supplement: Supplementary file 3 — Supporting information. [file HEX-28-e70228-s005.docx]

TOPIC GUIDE

**Stakeholder FGDs with implementors: Year 2**

**Research questions:**

1. How have you gone about moving the community to be recruited and retained?
   1. Awareness of CMCs and their activities
      - - Resonates with the community, leading to wanting to be recruited
        - Health literacy demonstrates benefits of health gains to improve self-motivation and outreach
        - Intent to join activities
   2. Adoption of programme
      - - Going to health literacy activities
        - Doing new health behaviors
   3. Advocacy for attending existing activities/programmes
2. Regarding volunteers, how has capacity (volume) and their capability training (skill growth) been built?
   1. Awareness of CMCs and their activities
      - - Intent to volunteer
   2. Adoption of volunteerism
      - - Opinion leaders recruited
   3. Advocacy from residents/opinion leaders, creating their own outreach and activities. And ultimately advocacy for movement building as a model.

**Icebreaker**

Let’s talk about adoption of the programme. Take a minute to think about what you think is the most important behaviour change that you have observed in those taking part in your activities [in digital space].

Please write this on a piece of paper and we will discuss it as a group.

1. **How have you gone about moving the community to be recruited and retained?**
2. Awareness of CMCs and their activities

- How are you making the community aware of what movement building means?
  - In what ways do you think this positioning will resonate with the community?
  - How are you sharing the M4H brand? [Logo]
- What activities relating to health literacy have you undertaken? In what ways is this encouraging self-motivation for health behavior change?
- What kind of person might want to join with a little persuasion [hard to reach populations], but not immediately interested in taking part? What kind of things might be persuasive to them?

1. Adoption of programme

- How has your outreach been so far?
- Building on what you shared earlier, can you share specifically about the role of health literacy in forming new habits?
  - For example, related to having
    - A better social life and relationships with others
    - Improving psychological and emotional wellbeing [self-care and mental health]
    - Improving lifestyle and physical health

1. Advocacy for attending existing activities/programmes

- What types of advocacy for attending existing activities/programmes have you been seeing on the ground? -> interested to run their own activities

1. **Regarding volunteers, how has capacity (volume) and their capability training (skill growth) been built?**
2. Awareness of CMCs and their activities

- How are you creating awareness for volunteerism and engaging people’s interest in being a volunteer?

1. Adoption of volunteerism

- Have you managed to recruit new volunteers since we last spoke/since starting your programme? Please explain your answer and why/why not you think attracting new volunteers is working.
  - How has the coaching/training events with Bold or NHG, acting as community coaches been going?
  - Otherwise, how have you been training your volunteers?
- Can you tell us more about volunteer profiles? Who and why people are keen to volunteer?

1. Advocacy from residents/opinion leaders, creating their own outreach and activities. And ultimately advocacy for movement building as a model

- Have you observed residents/opinion leaders keen to create their own outreach and activities.
  - If not, how do you think you will encourage such a process in future.
  - If so, how has this come about?

**Anything to add**

- Can you summarize your biggest takeaway from this session?
- Do you have anything to add?

*The End.*

Alternative model of scaling up, where it becomes more about building an existing volunteering base and is less about the place based nature. “Losing your brand identity, but having community leaders and volunteers resonant with what you are doing”, leveraging existing pools of volunteerism/community leaders; rather than building new pools – for digital CMCs/less place-based CMCs (different models for different place-based).

Three channels of volunteers (1) organization volunteers; (2) self-initiated, not living in the neighborhood; (3) place-based, living in the neighborhood -> (2) and (3) resonate with the programme’s mission and activity/place-based nature -> (1) then to be short-term; while (2) and (3) then to be long-term volunteers [building up volunteer bases to avoid burnout]

Framing different models of scaling up for movement building theory
