# Supplementary material for: Defining Health Movements and Health Needs Across the Life Course: A Qualitative Study
Source: Health Expect. 2025 Apr 10;28(2):e70228. doi: 10.1111/hex.70228 (PMC11983323; doi:10.1111/hex.70228)
Supplement: Supplementary file 4 — Supporting information. [file HEX-28-e70228-s004.docx]

TOPIC GUIDE

**Go-along interviews: Process evaluation Year 1**

**Broad research questions:**

1. How can the key actors in the ecosystem best mine and intervene on the resonant narratives within the community?
   1. What implementation processes are likely to work – building in IRs.
   2. What is the role of technology, including overuse or problematic use of digital platforms as well as the benefits that they may provide?
2. How are M4H interventions proposing to drive participatory community approaches to health behavior change and change narratives?
   1. How do participants see of the “story” of the CMC– the mission / point of jointing the start-up as actors co-creating content

**Procedures:**

- Prior to the interview the researcher will have briefed and participant and introduced informed consent over the phone. Consent will be fully administered and obtained on the day.
- Organize to take a walk through the neighborhood where M4H activities are planned or where the participant thinks activities should be planned. The Interviewee will guide the walk, while the interviewer will ask the questions as below. The guide is semi structured so the questions can be asked in any order.
- The walk will take about 40 minutes to 1 hour, or longer if the person so chooses.
- The interviewer and interviewee can sit somewhere beforehand, such as a void deck or community café to clarify any questions relating to the informed consent and collect the participant’s signature. Equally at that time a short socio-demographic form will be completed.

**Basic introductory script:**

*To recap what will take place today, I’ll let you lead the walk and we can also discuss about how you find taking part in [insert programme name] so far and what you think will be good to do in the neighbourhood beyond what is already happening with programme activities.*

*Also, we just want to learn about your neighbourhood and what you like to do around here, and what types of things contribute to a healthy neighbourhood in your opinion. I have a recorder which I’ll keep recording our conversation on as we walk, with your permission. Please feel free to stop and sit and take me wherever you think is nice to explore. I’ll ask you questions about the walk you chose, what activities you like to do to improve health, feel good and have fun. By health we mean anything that contributes your taking part in social activities and which contributes to mental wellbeing as well as physical health. Also, how you think COVID-19 is changing the things we can do together. I can first introduce myself a little and then we can fill out a short socio-demographic form so I can keep a record with the interview.*

**The neighbourhood and M4H activities**

**Boxed topics were used in the present analyses.**

1. **EMOTIONAL RESONANCE - maintaining positioning consistent with what inspires the community and aligning branding**

- Tell me about where you want to take me and why you chose this walk?
- How does the programme you are taking part in use the spaces in your community?
- What about the programme makes you feel good about being part of it?
- Are you aware of Movement’s for Health and what it aims to do? Can you tell me what you think of getting the community to work together to motivate themselves to be healthier?
- Do you think the types of things on offer in the current programme are changing people’s behavior?
  - Why or why not? In what ways?
- Can you tell me about how people of the age or ages targeted by the programme tend to remain healthy in your neighborhood?
- How do you think taking part in programme activities will improve community health, particularly at the stage of life we are asking about?
- How has factoring in planning and preparations around COVID-19 affected things?

1. **PARTICIPATORY ENGAGEMENT**

- How can people be more involved in choosing how the programme will run and what it will look like?
  - How about would community actors be identified and persuaded to be included in deciding how to set up activities? Perhaps as volunteers?

1. **OUTREACH AND GETTING STARTED**
2. **Demand generation**

- What kind of outreach works best to get people interested in taking part in things [as beneficiaries]? What keeps them coming back [retention]?
- What kind of person might want to join with a little persuasion, but currently isn’t taking part?
  - What kind of things might be persuasive to them?

1. **CAPACITY AND CAPABILITY– growing volunteer pools and knowledge**

- Do you think it is feasible run activities where participants “pass on” or teach about things they learnt to others which they learnt through the programme? Why/why not?
- What could make people of your life stage more involved, even keen to run their own activities in the programme?

1. **BUILDING PARTNERSHIPS**

- Are there any other partnerships with organizations, experts you can think of that would help to enhance the programme? in what ways?
- Are any such collaborations currently happening? If so, what do you think of them?
- How can these be encouraged?

**Potentially relevant topics reviewed to supplement present analyses.**

1. **ROLE OF TECHNOLOGY AND SMART PHONE USE**

- During covid-19 we started using Zoom, WhatsApp etc. a lot more to stay digitally connected. Can you tell me about your own use of digital platforms?
- In what ways is your use of such platforms problematic to you or beneficial (probe on mental health / anxiety), if either?
- How does taking part in the current community programme affect your smart phone use?
- What are your views on how smartphone can be problematic in the community?
- What solutions do you see to help draw people away from being too digitally focused and interact more in the community / attend the activities offered by the programme?
- Do you see any opportunities to help people that have issues / or are ‘addicted to smart phone use’?

***ANYTHING TO ADD**

- Do you have anything to add?

*The End.*
