# Supplementary material for: Defining Health Movements and Health Needs Across the Life Course: A Qualitative Study
Source: Health Expect. 2025 Apr 10;28(2):e70228. doi: 10.1111/hex.70228 (PMC11983323; doi:10.1111/hex.70228)
Supplement: Supplementary file 5 — Supporting information. [file HEX-28-e70228-s001.docx]

**Development and Evaluation of the Movements for Health (M4H) Programme: E-diary Activity**

| **Participant’s Name:** | **Date:** |
| --- | --- |
| **Pseudonym:** | **CMC Name:** |
| **Role in Programme:** |  |

**Entry 1: What is your story?**

**What does being healthy mean to you?**

**Entry 2: Social Health**

**What kinds of relationships/friendships do you have at [CMC name]? What do you like about them? Share at least one story with us.**

**Entry 3: Psychological Health**

**Tell us about some emotions you have experienced since joining [CMC name]. Are they good or bad emotions? Why do you feel this way? Share at least one story with us.**

**Entry 4: Biological Health**

**Has your physical health changed since joining [CMC name]? Does your body feel better or healthier? Share at least one story with us.**

**Entry 5: Media**

**Please share all media uploads here.**

|  |
| --- |
