# Supplementary material for: Defining Health Movements and Health Needs Across the Life Course: A Qualitative Study
Source: Health Expect. 2025 Apr 10;28(2):e70228. doi: 10.1111/hex.70228 (PMC11983323; doi:10.1111/hex.70228)
Supplement: Supplementary file 6 — Supporting information. [file HEX-28-e70228-s002.docx]

Overview of Movements for Health (M4H) Community Movement Champion Programme Content

| CMC | Target Audience | Goal |
| --- | --- | --- |
| (A) | Children and Youth | Empowering children and youth, through the use of a community kitchen and garden, to make better food choices. |
| (B) | Children and Youth | Equipping children and youth with reading literacy to make informed choices regarding food, mental wellbeing, sleep and dental hygiene. |
| (C) | Children and Youth | Working through public educators, adolescents are empowered to make better dietary choices. |
| (D) | Intergenerational | A multi-behavioral programme, adopting a participatory approach, where programme participants are asked to rank and choose which aspect of health (i.e. healthy eating, physical exercise, mental health, social connectedness, preventative health) concerns them most and in tandem enabling them to address it. |
| (E) | Seniors | Raising awareness around sarcopenia and encouraging the adoption of physical exercise, through podcasts, radio shows and in-person events held at local Active Ageing Centers and Community Centers. |
| (F) | Seniors | Promoting active ageing and combatting social isolation in seniors through walking trails. A secondary goal is to generate awareness around environmental sustainability, with cues to action. |
| (G) | Seniors | Combatting social isolation in seniors through the creation of an online forum via Facebook, with the goal of encouraging both on and offline social connections at the neighborhood level. |
